# Localizing Transcriptional Regulatory Elements at the Mouse *Dlk1* Locus

**DOI:** 10.1371/journal.pone.0036483

**Published:** 2012-05-11

**Authors:** Eric D. Rogers, Jenniffer R. Ramalie, Erin N. McMurray, Jennifer V. Schmidt

**Affiliations:** The Department of Biological Sciences, University of Illinois at Chicago, Chicago, Illinois, United States of America; CNRS, France

## Abstract

Much effort has focused recently on determining the mechanisms that control the allele-specific expression of genes subject to genomic imprinting, yet imprinting regulation is only one aspect of configuring appropriate expression of these genes. Imprinting control mechanisms must interact with those regulating the tissue-specific expression pattern of each imprinted gene in a cluster. Proper expression of the imprinted *Delta-like 1* (*Dlk1*) - *Maternally expressed gene 3* (*Meg3*) gene pair is required for normal fetal development in mammals, yet the mechanisms that control tissue-specific expression of these genes are unknown. We have used a combination of *in vivo* and *in vitro* expression assays to localize *cis*-regulatory elements that may regulate *Dlk1* expression in the mouse embryo. A bacterial artificial chromosome transgene encompassing the *Dlk1* gene and 77 kb of flanking sequence conferred expression in most endogenous *Dlk1*-expressing tissues. In combination with previous transgenic data, these experiments localize the majority of *Dlk1 cis*-regulatory elements to a 41 kb region upstream of the gene. Cross-species sequence conservation was used to further define potential regulatory elements, several of which functioned as enhancers in a luciferase expression assay. Two of these elements were able to drive expression of a *lacZ* reporter transgene in *Dlk1*-expressing tissues in the mouse embryo. The sequence proximal to *Dlk1* therefore contains at least two discrete regions that may regulate tissue-specificity of *Dlk1* expression.

## Introduction

Multicellular organisms generate an enormous diversity of distinct cell types during embryonic development by the spatial and temporal regulation of gene expression. Much of this regulation occurs at the level of transcription, where positive and negative c*is*-regulatory elements are employed to achieve fine control over expression. Enhancers and silencers are DNA elements composed of binding sites for transcription factors that interact in *cis* with promoter-bound proteins to control gene expression, functioning to allow or prevent, respectively, interaction with the promoter-proximal transcriptional machinery. Many developmentally important genes are also regulated by genomic imprinting, the parental-specific expression of the two alleles of an autosomal gene [1]. Imprinted gene expression is controlled on multiple levels by DNA CpG methylation, histone protein modification, higher order chromatin packaging, and likely additional unknown processes. These imprinting regulatory mechanisms are believed to specify the on or off state of each allele of an imprinted gene, with the pattern and levels of expression of that gene determined by tissue-specific enhancers. The imprinting regulatory machinery must therefore coordinate with the transcriptional regulatory machinery, and understanding the control of imprinted gene expression requires full knowledge of both components.

The imprinted *Dlk1*-*Meg3* locus on mouse chromosome 12 (human 14q32) contains maternally- and paternally-expressed genes, of which *Dlk1* and *Dio3* are the only members to produce unique protein products [2]–[4]. *Dlk1* encodes a transmembrane protein containing six epidermal growth factor-like motifs with similarity to the Delta-Notch-Serrate family of signaling molecules [5], [6]. The full length Dlk1 protein is cleaved to produce membrane-bound and secreted/soluble forms of the protein by the tumor necrosis factor alpha converting enzyme (TACE) [7]. Conflicting data exist as to the roles of the membrane-bound and soluble forms of the Dlk1 protein, which appear to play complex and perhaps complementary roles in multiple differentiating cell types [6], [8], [9], [10]. Several studies have shown that Dlk1 can act as a Notch antagonist, a role consistent with its lack of the conserved DSL interaction domain found in other Notch ligands [11]–[14]. In cultured cells, soluble Dlk1 has been shown to inhibit the ability of Notch to transactivate target genes such as *Hes1* [12], [13]. Additionally, Dlk1 appears to activate the insulin/IGF-I signaling pathway, leading to activation of the extracellular regulated kinase/mitogen-activated protein kinase (ERK/MAPK), but may also signal through non ERK-dependent mechanisms [15]–[17].

*Dlk1*-null mice display pre- and postnatal growth retardation, partially penetrant neonatal lethality, malformation of the ribs and sternum and altered lipid metabolism [18]. Overexpression of *Dlk1* is also deleterious, as animals expressing a two-fold excess of *Dlk1* showed overgrowth during embryogenesis and postnatal lethality possibly due to feeding defects [19]. Animals expressing a three-fold *Dlk1* excess died during late gestation with severe edema, skeletal defects and underdeveloped lungs. These opposing animal models bear many similarities to the phenotype of human patients with either maternal or paternal uniparental disomy for chromosome 14, which results in a loss or increase of *Dlk1* expression, respectively [20].

Functionally, Dlk1 is best understood as a regulator of the transition of preadipocyte cells to mature adipocytes, and it is downregulated upon the onset of differentiation in cultured preadipocyte cells and in differentiating mesenchymal stem cells [6], [21]–[23]. Dlk1 also plays a role in skeletal muscle development and regeneration, and mice with a myoblast-specific deletion of *Dlk1* are smaller than normal, with decreased skeletal muscle mass [24]. These animals also show impaired regeneration of adult muscle after injury, when satellite cells must be stimulated first to proliferate and then to differentiate into new and engrafting myoblasts.

*Dlk1* and *Meg3* display complex patterns of tissue- and temporal-specific regulation. *Dlk1* is expressed in the placenta and many embryonic tissues, and expression is generally concordant between mouse and human. During development, *Dlk1* is expressed in fetal hepatocytes, lung epithelium, adrenal cortex, the chondroblasts of the developing skeleton, developing skeletal myoblasts, the ventral diencephalon and Rathke’s pouch, peripheral nerves prior to myelination, bone marrow and the thymic epithelium [25]–[31]. In the pancreas, *Dlk1* is initially expressed in most cells of the pancreatic rudiment, becoming restricted first to the differentiating endocrine cells, and finally to the beta cells alone [32]. In the extraembryonic tissues of the mouse, *Dlk1* is expressed in the fetal endothelium of the yolk sac and the placental labyrinth [27], [33]. *Dlk1* expression declines after birth, and in the adult becomes restricted to the pancreatic beta cells, the pituitary somatotrophs, the bone marrow, and the zona glomerulosa of the adrenal gland [25], [28], [31], [34], [35]. Previous groups have identified general positive and negative transcriptional elements within the *Dlk1* promoter-proximal region, but nothing is known about the distal elements that govern the complex tissue- and temporal-specific expression pattern of this gene [36]–[39].

Closely linked to *Dlk1* is the maternally expressed *Meg3* gene, which produces a long noncoding RNA that may function as a developmental regulator and/or tumor suppressor [2], [3], [40]–[42]. *Meg3* is expressed in the preimplantation mouse embryo, and as differentiation proceeds it is found in the developing pancreas, the paraxial mesoderm and the resulting myoblasts, the ventral diencephalon and Rathke’s pouch, and in the dorsal root ganglia, spinal cord and forebrain [43], [44]. In mouse extraembryonic tissue, *Meg3* is expressed in the yolk sac, the labyrinthine trophoblast, and some labyrinth endothelial cells [33], [43]. Comparison of *Dlk1* and *Meg3* expression shows a complex pattern of both shared and discrete cell type-specific expression. Both genes are strongly expressed in the myotome and migrating myoblasts, for example, and both are down-regulated in skeletal muscle postnatally [27], [33]. In the pituitary, however, *Dlk1* and *Meg3* show coordinate expression in the infundibulum and Rathke’s pouch during development, but *Dlk1* subsequently becomes restricted to somatotrope cells, while *Meg3* is expressed only in gonadotropes [27], [33].

Bacterial artificial chromosome (BAC) transgenesis in the mouse is often used to localize regulatory elements within the sequences surrounding a genomic locus [45]. Large BAC transgenes can recapitulate genomic imprinting as well as expression, making them ideal for studying imprinted gene regulation. Previous work from our laboratory showed that the *Meg3* gene was expressed and properly imprinted from the *28G5* BAC transgene that spans the region from 3.5 kb upstream of *Dlk1* to 69 kb downstream of *Meg3,* however, *Dlk1* was not expressed from this transgene [46]. Another group subsequently showed that *Dlk1* is expressed in a subset of its normal pattern from a BAC transgene carrying 49 kb of sequence upstream of *Dlk1* (*Tg^Dlk1−70^*), but not from a BAC transgene carrying only 8 kb of upstream sequence (*Tg^Dlk1−31^*) [19]. In the current study we use additional transgenic and *in vitro* approaches to identify discrete regions of sequence proximal to *Dlk1* that contain *cis*-regulatory elements capable of directing *Dlk1*-like expression in the mouse embryo.

## Materials and Methods

### Generation of 127H5 BAC Transgenic Mice

The 127H5 BAC clone was isolated from the CITB 129/Sv mouse genomic library (Children’s Hospital Oakland Research Institute). The clone was end-sequenced and found to span 84 kb, from 41 kb upstream to 36 kb downstream of the *Dlk1* gene. The BAC clone was linearized at a unique *Cla*I site 10.5 kb upstream of *Dlk1*, and injected into fertilized FVB/N embryos as described previously [46]. The resulting offspring were genotyped by PCR using the primers OL477, 5′-GCTTGAGTATTCTATAGTGTCA-3′, and OL478, 5′-CAACGCAATTAATGTGAGTTAG-3′, which amplify a 170 bp fragment of the BAC vector. Founder animals were bred to wild type FVB/N mice to establish independent transgenic lines, and transgene copy number was calculated by Southern blotting as described [46].

### Expression and Imprinting Analysis

*Dlk1* expression was analyzed by Northern blotting, and imprinting analysis was performed using RT-PCR and direct sequencing as described [2]. Embryos and placentae were recovered between e12.5 and e14.5 and adult tissues were obtained from 3–4 week old animals. Total RNA was purified using LiCl-urea precipitation [47]. For Northern analysis, 10 µg of total RNA was separated on 1% formaldehyde agarose gels and transferred to Hybond N+ membranes. Membranes were hybridized with *Dlk1* and *β*-*actin* probes; the *Dlk1* probe is a 735 bp fragment corresponding to nucleotides 685 to 1420, and the *β-actin* probe is a 1200 bp fragment spanning exons 3–7. Hybridization was carried out using Express-Hyb (Clontech) overnight at 65 ^o^C, and the membrane was washed three times for 15 min at 50°C with Northern wash I (2×SSC and 0.05% SDS), and twice for 15 min at 50°C with Northern wash II (0.1×SSC and 0.1% SDS). The membrane was exposed to a phosphorimager screen or film for analysis and *Dlk1* signal intensity was normalized to *β-actin* expression. A student’s two-tailed t-test was used to determine statistical significance. For RT-PCR, 2 µg total RNA was reverse transcribed using Superscript III (Invitrogen) and oligo-dT, RT reactions were diluted 1∶10 and 2 µl was used for all PCR analyses.

### Identification and Cloning of Conserved Elements Upstream of Dlk1

Sequence conservation was analyzed using the University of California Santa Cruz Genome Browser (http://genome.ucsc.edu/) to compare the genomes of *Mus musculus* (July 2007 assembly), *Rattus norvegicus* (Nov. 2004), *Homo sapiens* (Feb. 2009), *Pan troglodytes* (Oct. 2010), *Macaca mulatta* (Jan. 2006), *Pongo abelii* (July 2007), *Callithrix jacchus* (Mar. 2009), *Otolemur garnettii* (Dec. 2006), *Canis familiaris* (May 2005), *Felis catus* (Dec. 2008), *Equus caballus* (Sept. 2007), *Bos taurus* (Oct. 2007), *Dasypus novemcinctus* (May 2005) and *Loxodonta africana* (July 2009). Vista browser (http://genome.lbl.gov/vista) was also used to align *Mus musculus* (July 2007) and *Homo sapiens* (Feb. 2009) sequences downloaded from Ensembl (http://ensenmbl.org). Nine elements upstream of *Dlk1* (CE1–CE9) were selected that contained ≥50 bp stretches of DNA with ≥70% sequence homology. The *Dlk1* promoter [36], [37], [48] was isolated as a 256 bp *Xho*I-*Rsr*II restriction fragment and cloned into the *Xho*I site of the pGL3-B plasmid (Promega), generating plasmid pGL3-*Dlk1*P. The conserved elements were amplified by PCR from 127H5 DNA using primers listed in Table 1; PCR conditions for all primers were 94°C, 30 sec, 60°C, 1 min and 72°C, 1 min, for 35 cycles. The PCR products were cloned into pCRII-TOPO, then excised using *Eco*RI, blunt ended and inserted into the *Sma*I site of pGL3-*Dlk1*P. These plasmids were termed pGL3-CE, where CE stands for the conserved element.

**Table 1:** Primers used to amplify *Dlk1* upstream conserved elements.

Conserved element	Primer name	Primer sequence
CE1	OL1152	5′-TTAAGGAGGAGAGCCACTCACTGT-3′
	OL1153	5′-TCAGGCAAAGGCCAGAGACAGAAA-3′
CE2	OL1154	5′-CTGTTGCGATGTAACTAGGTGGGA-3′
	OL1155	5′-AAAGGCCAAGGACACCAGATCAGA-3′
CE3	OL1156	5′-TGGTTTGCCCTGCCTTCCTAGTAT-3′
	OL1157	5′-ATCTGAAGGGTGCCACAACTGTCT-3′
CE4	OL1158	5′-GCAGGGCTTCGTTCTTTCCATGTT-3′
	OL1159	5′-TCCATACATGGCCGGATGTGGTTA-3′
CE5	OL1101	5′-TCATCACCCAGCAAGAAGACAGGT-3′
	OL1102	5′-TCTGCTCAACCAGCCTAGCTTACT-3′
CE6	OL1090	5′-TAAGGCACTACAGCAAGGAAGCCA-3′
	OL1100	5′-TCCTGGGCATCAAACATGACCACT-3′
CE7	OL1093	5′-ACCTGAGGACGCCATTTGACCATA-3′
	OL1094	5′-GCTCGCCAGCCAGAAGTAGAATTT-3′
CE8	OL1095	5′-GCTCTGTGTGCAATCTGCTTTCCA-3′
	OL1096	5′-CAATGCCTGCAGCTTACCACACTT-3′
CE9	OL1097	5′-CCACACAACCTTCACCCAACCATT-3′
	OL1098	5′-TTACTGGCTAGGCTCACAGAGCAA-3′

### Cell Culture and Transfection

The C2C12 cells were maintained in high glucose Dulbecco’s Modified Eagle Medium (DMEM) (Invitrogen) with 10% fetal bovine serum (FBS) and 4 mM L-glutamine. NIH-3T3 cells were maintained in high glucose DMEM with 10% calf serum and 4 mM L-glutamine. SVR cells were maintained in high-glucose DMEM with 5% FBS and 4 mM L-glutamine. Y-1 cells were maintained in F-12K medium (Invitrogen) with 15% horse serum, 2.5% FBS and 2 mM L-glutamine. All cell lines were obtained from ATCC. All media contained 100 µg/ml penicillin/streptomycin and the cells were maintained in a humidified incubator at 37°C with 5% CO_2_.

For transfection, C2C12, NIH-3T3 and SVR cells were plated in 24-well plates at a density of 50,000 cells/well; Y-1 cells were plated at a density of 150,000 cells/well. Cell lines were transfected with 200 µl of serum-free media per well containing Transfast reagent at a 1∶1 ratio, 0.7 µg of the appropriate pGL3 DNA, and 0.3 µg of the pSV-βgal control vector. Growth media was replaced after 1 hour, and the cells were lysed 48 hours post-transfection with 150 µl of 1× Glo Lysis Buffer (Promega). Luciferase production was assayed using a Clarity Microplate Luminometer (Bio-Tek Instruments Inc.) by adding 50 µl of cell lysate to 50 µl of Steady-Glo Luciferase Assay Substrate (Promega). Expression of *lacZ* as β-galactosidase activity was measured by incubating 50 µl of cell lysate with 143 µl of Z-Buffer (0.06 M Na_2_HPO_4_, 0.04 M NaH_2_PO_4_, 0.01 M KCl, 0.001 M MgSO_4_), 36 µl ONPG (4 mg/ml in diH_2_0) and 1 µl 2–mercaptoethanol reagent (Fisher Scientific) at 37°C for 30 min. After incubation, 300 µl of diH_2_O was added to the reactions and the absorbance was measured at 414 nm. Luciferase expression was normalized to β-galactosidase activity for each well. A student’s two-tailed t-test was used to determine statistical significance.

### Generation of CE-lacZ Transgenic Mice

A 2.4 kb fragment containing two copies of the chicken β-globin insulator, 5′HS4 [49], was excised from plasmid pJC13-1 using *Eco*RI and *Bam*HI and cloned in duplicate into the *Bgl*II and *Hpa*I sites of the pSL1180 shuttle vector, generating plasmid pSL-I. An *Xba*I site in pNASS-β was destroyed to allow subsequent cloning, and the *lacZ* gene was excised from this plasmid using *Eco*RI and *Hind*III and inserted into the *Eco*RV site of pSL-I, generating pSL-IL. The *Dlk1* promoter was excised from pGL3-*Dlk1*P using *Xho*I and *Nhe*I and cloned into the *Nhe*I site of pSL-IL to generate pSL-ILP. The CE4, CE8 and CE9 fragments were excised from pCRII-TOPO using *Eco*RI and cloned into the *Xba*I site of pSL-ILP to generate CE4-*lacZ,* CE8-*lacZ* and CE9-*lacZ*, respectively. CE4-*lacZ* and CE8-*lacZ* were linearized using *Nco*I and *Sna*BI, CE9-*lacZ* was linearized using *Bst*BI and *Sna*BI, and all transgenes were purified with QIAEX II (Qiagen). Linearized DNA was injected into FVB/N embryos by the UIC Transgenic Production Service, and the embryos implanted in pseudopregnant ICR females. Embryos were collected at e13.5 and fixed in 4% paraformaldehyde for 90 min on ice, washed 3 times at room temperature (RT) for 30 min (2 mM MgCl_2_, 0.01% deoxycholic acid, 0.02% NP-40 in 1× PBS), and stained overnight in the dark at RT (1 mg/ml X-gal, 5 mM potassium ferricyanide, 5 mM potassium ferrocyanide, 2 mM MgCl_2_, 0.01% deoxycholic acid, 0.02% NP-40). The reaction was stopped by washing the embryos 3 times in 1× PBS at RT. Embryos were genotyped from yolk sac DNA using primers OL410, 5′-GGGGACGACAGTACGAAAAGGC-3′, and OL411, 5′-GTATCGGCCTCAGGAAGATCGC-3′ to generate a 789 bp *lacZ* product. PCR conditions were 94°C, 30 sec, 60°C, 1 min and 72°C, 1 min, for 35 cycles. Transgenic embryos were embedded in paraffin, and 8-micron sagittal sections were cut and counterstained with eosin. Low power images were collected on a Leica MZFLIII dissecting microscope using a Leica DFC320 camera. High power images were collected on a Zeiss Axiovert 200 M microscope using a Zeiss AxioCam MRc5 camera.

## Results

### Dlk1 is Expressed but not Imprinted from the BAC 127H5 Transgene

**Figure 1:**
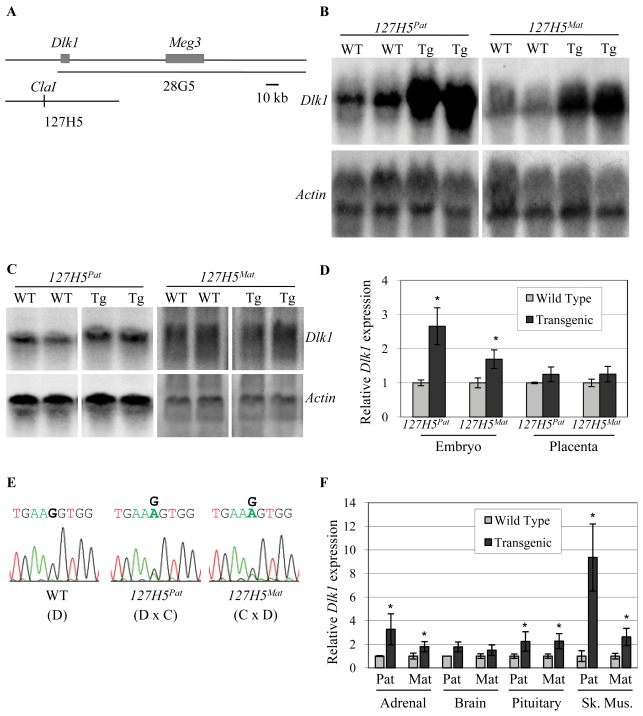
*Dlk1* is expressed but not imprinted from the 127H5 transgene. (A) Schematic of the *Dlk1*-*Meg3* BAC clones used to generate transgenic mice; the *28G5* transgene was described previously [46]. The 127H5 BAC was linearized at a unique *Cla*I site. (B, C) Representative Northern blots for *Dlk1* mRNA in midgestation wild type (WT) and heterozygous transgenic (Tg) embryo (B) and placenta (C), after paternal (*127H5^Pat^*) and maternal (*127H5^Mat^*) inheritance. The mouse *β-actin* gene was used as a loading control. (D) Quantitative Northern data for blots shown in B & C; expression is normalized to *β-actin*. Gray bars represent wild type samples and black bars represent *127H5* transgenic samples upon paternal (*127H5^Pat^*) or maternal (*127H5^Mat^*) transmission in crosses to Cg12. (E) Direct sequencing assay for *Dlk1* imprinting in wild type (WT) and heterozygous transgenic (Tg) F_1_ embryos after paternal (*127H5^Pat^*) and maternal (*127H5^Mat^*) inheritance. D indicates wild type animals carrying only the *M. domesticus* allele, while D×C or C×D indicates offspring of crosses to the Cg12 line carrying a *M. castaneus* allele, with the female genotype listed first. (F) Quantitative Northern blot analysis for *Dlk1* mRNA in 3–4 week old *127H5* tissues. Expression is normalized to *β-actin*, and each bar represents 8–10 animals. Gray bars represent wild type samples and the black bars represent *127H5* transgenic samples upon paternal (Pat) or maternal (Mat) transmission. In all figures asterisks indicate p≤0.05.

Previous work showed that *Dlk1* was not expressed from the *28G5* transgene spanning the mouse *Dlk1*-*Meg3* region, suggesting that elements outside of this BAC are required for *Dlk1* expression (Fig. 1A) [46]. To expand the search for *Dlk1* regulatory sequences, transgenic mice were produced using the overlapping BAC 127H5, which extends 41 kb upstream and 36 kb downstream of the mouse *Dlk1* gene (Fig. 1A). The BAC was linearized at a unique *Cla*I site at −10.5 kb relative to the *Dlk1* transcriptional initiation site. While this places much of the upstream sequence in a position downstream of *Dlk1* in the transgene, this is not likely to alter the function of tissue-specific enhancers. Enhancers are by definition position- and orientation-independent, a fact that has been exploited in a wide variety of heterologous *in vivo* expression vectors. We have also shown that enhancers directing tissue-specific expression of the linked *Meg3* gene function appropriately even when located on a separate, cointegrated DNA fragment [46].

Three founder animals were obtained that carried 8, 2, and 2 copies of the BAC (data not shown). Individual transgenic lines were established by breeding to FVB/N mice, and *Dlk1* expression levels were analyzed in whole embryo at embryonic day 12.5 (e12.5). Since the *Dlk1* gene is subject to genomic imprinting, expression levels were assayed in heterozygous embryos after both maternal and paternal transmission of the transgene. *Dlk1* expression from two of the lines was equal to wild type littermates by Northern blotting, suggesting that *Dlk1* was not expressed from these transgenes (data not shown). In our experience, the lack of expression from two of the three lines is not unusual for transgenic animals, in which integrated sequences may be silenced by virtue of their integration site, concatenated structure or bacterially-derived vector sequences. The third line, which carries 2 copies of the BAC, showed a 1.8 and 2.5 fold increase in *Dlk1* expression upon maternal and paternal transmission of the transgene, respectively (Fig. 1B). These data show that *Dlk1* is expressed from the transgene in this *127H5* line, but suggest that it is not imprinted (Fig. 1B, D). *Dlk1* is normally expressed in the mouse placenta at e12.5, but none of the *127H5* lines showed increased levels of *Dlk1* in placenta when compared to wild type (Fig. 1C, D and data not shown).

To more directly analyze imprinting of the *127H5* transgene, we took advantage of a congenic mouse line (*Cg12*) that carries the *Dlk1*-*Meg3* region of chromosome 12 from *Mus musculus castaneus* (C) on a *Mus musculus domesticus* (D) background [50]. The *Cg12* mice allow the use of sequence polymorphisms between C and D animals for imprinting analysis in F_1_ offspring. Analysis of embryos derived from a cross between *127H5* mice carrying D alleles, and *Cg12* mice carrying C alleles, showed that both parental alleles were expressed, and that *Dlk1* is not imprinted on the *127H5* transgene (Fig. 1E).

To determine the tissue-specific profile of *127H5 Dlk1* expression, three-week old *127H5* mice were analyzed for *Dlk1* mRNA levels in isolated adrenal gland, brain, pituitary gland and skeletal muscle (Fig. 1F). A significant increase in *Dlk1* expression over wild type was observed by Northern blotting in the adrenal and pituitary glands and skeletal muscle of *127H5* mice, but no increase in *Dlk1* expression was observed in brain (Fig. 1F). The levels of *127H5-*derived *Dlk1* expression were independent of the direction of parental transmission, consistent with the lack of imprinting observed in the embryo (Fig. 1E). Levels of *Dlk1* in skeletal muscle were increased to a greater degree than those seen in other tissues, indicating a strong skeletal muscle enhancer may lie within *127H5*, and/or that a silencer that modifies *Dlk1* expression is not present on the BAC. Skeletal muscle *Dlk1* levels were also higher in animals inheriting the transgene paternally than in those inheriting it maternally, despite the lack of overt imprinting of the transgene as a whole. While the reason for this expression difference is unclear, it is possible the transgene carries some component of the imprinting machinery that, in isolation, remains capable of driving increased paternal expression in certain tissues or in highly-expressing tissues. These data suggest that *127H5* contains the tissue specific elements required for *Dlk1* expression in some, but not all, sites of endogenous *Dlk1* expression.

These results are consistent with work from da Rocha et al, which showed that a BAC transgene carrying 49 kb of sequence upstream of *Dlk1* (extending 8 kb beyond the 5′ end of *127H5*) is active in many sites of endogenous *Dlk1* expression, including kidney, skeletal muscle and liver, but is not expressed in placenta [19]. Similarly to the data shown here, the *Dlk1* gene was not imprinted in the context of this transgene.

### Conserved Elements Upstream of Dlk1 Function as Enhancers in Cell Culture

**Figure 2:**
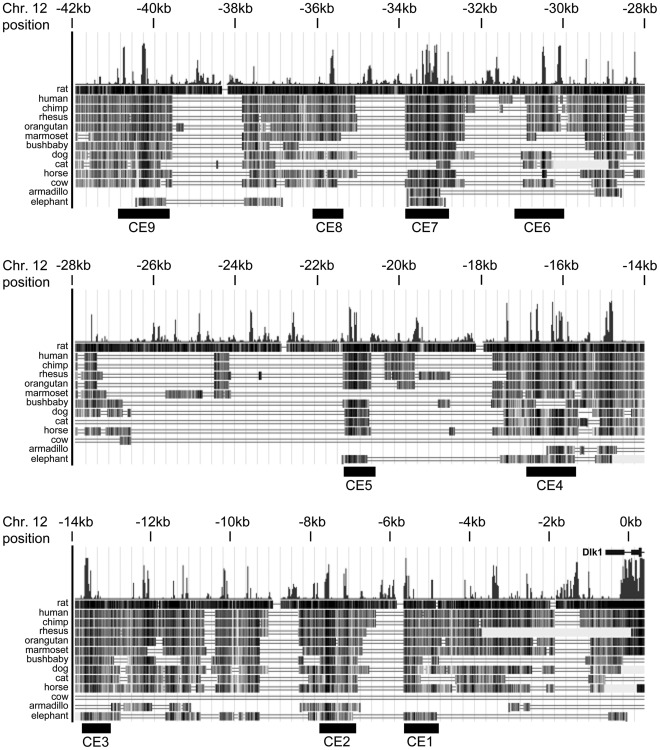
Sequence conservation in the *Dlk1* upstream region. The sequence upstream of *Dlk1* was analyzed for regions of conservation across multiple species. The conserved elements chosen for further analysis are numbered CE1 to CE9. The region being displayed corresponds to the July 2007 mouse genome assembly; assembly dates for other species are given in the Methods (adapted from the UCSC Genome Browser).

The data obtained from the *28G5* and *127H5* transgenes suggest that elements located in the non-overlapping sequence (−3.5 to −41 kb upstream of *Dlk1*) are capable of directing gene expression in multiple *Dlk1*-expressing tissues. The sequence upstream of *Dlk1* was analyzed for conserved regions across species using the University of California Santa Cruz (UCSC) Genome Browser (http://genome.ucsc.edu/) and the Vista browser (http://genome.lbl.gov/vista) (Fig. 2 and data not shown). The UCSC browser measures evolutionary conservation between multiple vertebrate species using *phastCons* and *phyloP*. Vista uses a combination of Shuffle-LAGAN global alignments and local BLAT alignments to construct genome-wide pairwise DNA alignments between two species. The UCSC Genome Browser alignment shown in Figure 2 makes use of all currently available genome sequence, and with one exception supports the original alignments performed in 2006. The highly conserved region at approximately −15 kb was not present in comparisons performed when fewer species were available, and awaits future functional analysis.

**Figure 3:**
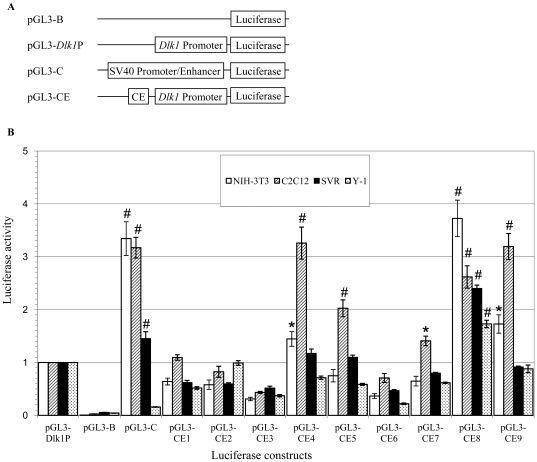
Transcriptional activation by conserved elements is cell line-dependent. (A) Luciferase expression plasmids used to assay enhancer function in cell culture. “*Dlk1* Promoter” signifies the *Dlk1* basal promoter and CE signifies the individual conserved elements tested. Plasmid pGL3-B contains the promoterless luciferase gene, pGL3-*Dlk1*P contains the endogenous *Dlk1* promoter upstream of luciferase and pGL3-C contains the SV40 promoter/enhancer upstream of luciferase. (B) Enhancer activity observed in the NIH-3T3, C2C12, SVR and Y-1 cell lines. Expression is normalized to pGL3-*Dlk1*P for each cell type; the results are presented as mean ± SEM. P-values relative to pGL3-*Dlk1*P expression are indicated by *, p≤0.05; #, p≤0.01 (n = 3) (Student’s t-test).

Nine conserved elements of approximately 1 kb (CE1–9), that contained ≥50 bp stretches of DNA with ≥70% sequence homology, were chosen for further analysis (Fig. 2 and data not shown). A luciferase reporter assay was used to screen the CEs for regulatory activity; CEs were cloned into a luciferase expression vector driven by the *Dlk1* promoter, generating plasmids pGL3-CE1 through pGL3-CE9 (Fig. 3A). Established cell lines selected from tissues that express the endogenous *Dlk1* gene: NIH-3T3 (mouse embryonic fibroblast), C2C12 (mouse myoblast), SVR (mouse pancreatic islet) and Y-1 (mouse adrenal tumor), were transiently transfected with the plasmids pGL3-B, pGL3-C, pGL3-*Dlk1*P, or the individual pGL3-CEs. Luciferase activity was measured relative to that driven by the *Dlk1* promoter alone, and all values were normalized to a transfection control (Fig. 3B). Multiple CEs functioned as enhancers in the luciferase assay, with CE4, 5, 7 and 9 showing higher levels of luciferase expression in C2C12 cells than in other cell types, suggesting that these elements might contain muscle specific enhancers (Fig. 3B). CE8 directed high levels of luciferase expression in multiple cell lines, suggesting that CE8 contains multiple enhancers or an enhancer that can function in multiple tissues (Fig. 3B). Expression plasmids carrying CE1, 2, 3 and 6 produced little or no luciferase activity over the *Dlk1* promoter alone.

### Conserved Elements can Direct lacZ Expression in vivo

**Figure 4:**
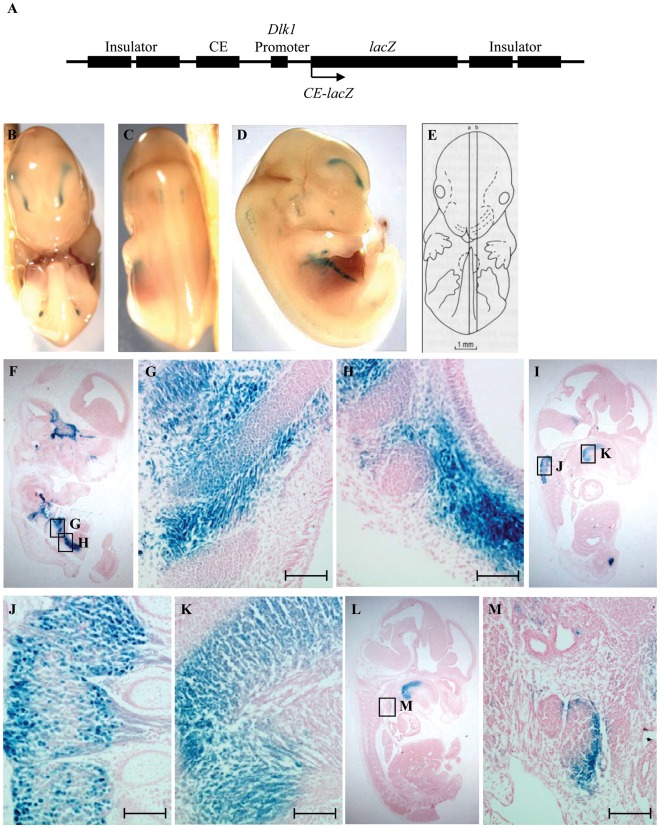
*CE4-lacZ* displays expression in a subset of *Dlk1*-expressing tissues. (A) Diagram of *lacZ* expression constructs used to produce *CE4-lacZ* and *CE8-lacZ* transgenic embryos. The arrow represents the direction of transcription, and CE stands for conserved element 4 or 8. (B–D) Ventral, dorsal and lateral views, respectively, of a representative whole mount transgenic embryo at e13.5. (E) Schematic representation of sagittal section planes used in these images [64]. (F–M) Sagittal sections of embryos under low magnification (F, I, L) and high magnification (G, H, J, K, M). All sections are oriented with anterior on top and dorsal to the left. Expression was seen in (F–H) intercostal muscle, body wall muscle, and ribs; (I, J) dorsal root ganglia; (I, K) intrinsic tongue muscle; (L, M) thymus. The embryo shown in (F) displays *lacZ* expression in the pituitary gland and trigeminal ganglion, both sites of endogenous *Dlk1* expression, but this pattern was not seen in other embryos carrying *CE4-lacZ*. Scale bars represent 100 µm.

As CE4, CE8 and CE9 conferred the highest level of enhancer activity in the reporter assay, these three regions were selected for *in vivo* analysis. Reporter transgenes were generated carrying each conserved element, and the *Dlk1* promoter, upstream of a *lacZ* cassette (*CE4-lacZ*, *CE8-lacZ*, and *CE9-lacZ*), and the transgenes were flanked by insulator elements to reduce position effects (Fig. 4A) [49]. The transgenes were injected into FVB/N fertilized eggs, and F_0_ embryos were collected at e13.5 for whole-mount analysis of *lacZ* expression. A total of 51 embryos were obtained from the *CE4-lacZ* injections, of which 5 carried the transgene and 4 showed *lacZ* expression. A total of 77 embryos were obtained from the *CE8-lacZ* injections, of which 16 embryos carried the transgene and 9 showed *lacZ* expression. A total of 45 embryos were obtained from the *CE9-lacZ* injections, of which 7 carried the transgene and 5 showed *lacZ* expression. Positively staining embryos were embedded and sectioned for histological analysis.

The chicken β-globin insulators functioned well in this system, as most embryos carrying a particular transgene showed similar patterns of expression despite their unique integration sites. In previous work from our laboratory, the β-globin insulators were shown to block the effects of virtually all external regulatory elements, and to confer no activity on their own (Steshina & Schmidt, unpublished). A particular tissue was scored as positive for *lacZ* expression only if it was found in 3 or more embryos for a given transgene. Embryos inheriting the *CE4-lacZ* transgene displayed *lacZ* expression in multiple regions of the musculoskeletal system and in some neuronal tissues (Fig. 4) (Table 2). *CE4-lacZ* expression was seen in the intercostal muscles, the epiphyses of the ribs and the body wall muscles of the abdomen (Fig. 4B–D, F–H). Expression was also found in the intrinsic muscles of the tongue (Fig. 4I, K). The expression seen in the dorsal root ganglia was present in the cell bodies as well as the axons (Fig. 4I, J). *CE4-lacZ* expression was also seen in the cortical region of the thymus (Fig. 4L, M).

**Figure 5:**
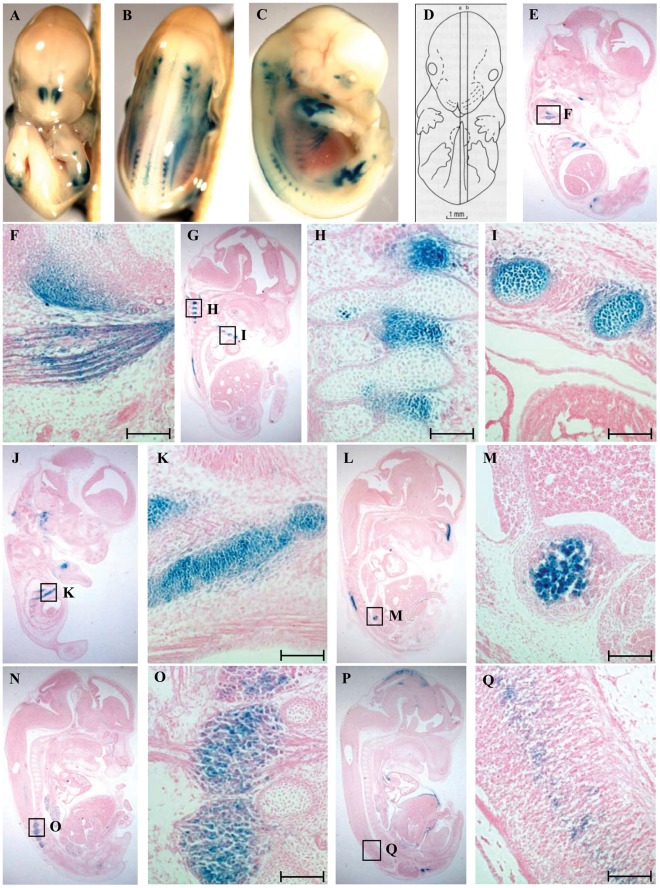
*CE8-lacZ* displays expression in a subset of *Dlk1*-expressing tissues . (A–C) Ventral, dorsal and lateral views, respectively, of a representative whole mount transgenic embryo at e13.5. (D) Schematic representation of sagittal section planes used in these images [64]. (E–Q) Sagittal sections of embryos under low magnification (E, G, J, L, N, P) and high magnification (F, H, I, K, M, O, Q). All sections are oriented with anterior on top and dorsal to the left. Expression was seen in (E, F) skeletal muscle of the limb, (G, H) intervertebral cartilage; (G, I–K) ribs and costosternal junctions; (L, M) chromaffin cells of the adrenal gland; (N, O) dorsal root ganglia; (P, Q) spinal cord. Scale bars represent 100 µm.

Embryos inheriting the *CE8-lacZ* transgene showed expression in the migrating skeletal muscle of the limbs (Fig. 5A–F), and in the developing skeleton including the ribs, costosternal junctions, intervertebral cartilage and vertebrae (Fig. 5B, C, G–K) (Table 2). *CE8-lacZ* expression was also observed in a subset of neuronal and neuroendocrine tissues, including the chromaffin cells of the adrenal medulla (Fig. 5L, M), the dorsal root ganglia (Fig. 5N, O), and the spinal cord (Fig. 5P, Q). No consistent pattern of expression was seen among the *CE9-lacZ* embryos, and so this element was not analyzed further. Overall, expression in the musculoskeletal system predominated from both the CE4 and CE8 elements, consistent with the high-level expression seen in muscle in the *127H5* transgenic mice, and the high C2C12 activity in the luciferase expression system. The pattern of expression from the *CE4-lacZ* and *CE8-lacZ* transgenes overlaps significantly with that of the endogenous *Dlk1* gene [27], [33], suggesting *Dlk1*-specific *cis*-regulatory elements are contained within CE4 and CE8. Despite this significant muscle expression, not all developing muscle is positive for *lacZ*, suggesting the presence of elements that direct *Dlk1* expression in a specific subset of muscle cells or at a specific stage of muscle development.

**Table 2:** Embryonic expression of *CE4-lacZ*, *CE8-lacZ* and *Dlk1.*

Tissue/organ	*CE4-lacZ*	*CE8-lacZ*	*Dlk1*
Tongue muscle	+	–	+
Intercostal muscle	+	–	+
Body wall muscle	+	–	+
Limb muscle	–	+	+
Intervertebral cartilage	–	+	+
Ribs	+	+	+
Thymus	+	–	+
Pituitary gland	–	–	+
Adrenal gland	–	+	+
Liver	–	–	+
Pancreas	–	–	+
Lung	–	–	+
Spinal cord	–	+	–
Dorsal root ganglia	+	+	–

+ indicates expression; – indicates no expression.

## Discussion

### The 127H5 Transgene Recapitulates Endogenous Dlk1 Expression

Previous work from our laboratory showed that the mouse *Meg3* gene was expressed in a subset of its endogenous pattern from the *28G5* transgene, and was imprinted in those tissues where it was expressed [46]. *Dlk1* was not expressed from *28G5* in any tissues, suggesting that *cis*-regulatory elements beyond the extent of 28G5 are required for *Dlk1* expression. Consistent with this idea, a subsequent study showed that the *Tg^Dlk1−70^* BAC transgene containing 49 kb of sequence upstream of *Dlk1* could direct *Dlk1* expression in a subset of endogenous *Dlk1*-expressing tissues [19] . The more proximal *127H5* transgene described here expands and refines this work, localizing multiple cis-regulatory elements that may control *Dlk1* in developing skeletal muscle, and in neuroendocrine tissues such as the pituitary and adrenal glands, to a 41 kb region upstream of *Dlk1* (Fig. 1). The similarities in expression between the *127H5* and *Tg^Dlk1−70^* BAC transgenes, and the lack of expression from *Tg^Dlk1−31^*, suggest most upstream regulatory elements lie within the −41 kb to −8 kb region.

*Dlk1* is not imprinted in the context of the *127H5* transgene; this was not surprising, as this transgene lacks both the intergenic and *Meg3* differentially methylated regions shown to be required for proper imprinting of the *Dlk1-Meg3* locus (Fig. 1E) [51]–[53]. In addition to expression in the embryo, placental expression of *Dlk1-Meg3* is also of interest, as many imprinted genes appear to play important roles in placental development and function [54]. *Meg3* is expressed in the placenta from the *28G5* transgene, but *Dlk1* does not show placental expression from any of the BAC transgenes, indicating yet more distal control elements exist for *Dlk1* extraembryonic expression [46] (Fig. 1C, D). We showed previously that 28G5 carries some *Meg3* control elements, and have now shown that *127H5* carries some *Dlk1* control elements; this gene pair therefore has a complex regulatory domain spanning more than 200 kb of sequence.

### Coordinate versus Independent Regulation of Dlk1 and Meg3

*Dlk1* and *Meg3* demonstrate a significant degree of overlap in tissue-specific expression during development, but within a single tissue these two genes are often expressed in dynamic and unique patterns in different cell types. Both genes are highly expressed in developing skeletal muscle, and they are coexpressed in fetal hepatocytes and in the early pancreas. During later pancreas development, however, *Meg3* expression is downregulated, while *Dlk1* retains high level expression but becomes restricted to the insulin-producing beta cells. Both genes are expressed in the developing pituitary gland, initially in the infundibulum that gives rise to the posterior lobe, and in Rathke’s pouch that gives rise to the anterior lobe. In adults, however, *Dlk1* expression is restricted to the anterior somatotrophs, while *Meg3* is expressed primarily in gonadotrophs [27], [33]. *Dlk1* is highly expressed in the lung, where *Meg3* is not expressed, and *Meg3* exhibits widespread expression in the central nervous system, while *Dlk1* is localized to a few brain regions where *Meg3* is absent [33]. While other imprinted gene pairs such as *H19-Igf2* share common regulatory elements, the limited overlap in cellular expression of *Dlk1-Meg3* suggests that the many of their *cis*-regulatory elements are unique to each gene. Data from the *28G5* and *127H5* transgenes largely support this hypothesis, with *Meg3* enhancers within *28G5* failing to activate *Dlk1*, while most *Dlk1* enhancers lie within the nonoverlapping region of *127H5*. Interestingly, the *CE4-lacZ* and *CE8-lacZ* transgenes directed expression in a subset of neuronal tissues where *Meg3*, but not *Dlk1*, is expressed, including the dorsal root ganglia (Fig. 4I–J, 5N–O) and the spinal cord (Fig. 5P–Q). Some *Meg3*-specific enhancers may therefore lie upstream of *Dlk1*, but are prevented *in vivo* from activating expression of *Dlk1*.

### Dissecting the Dlk1 Upstream Region

Multi-species comparison of the *Dlk1* upstream region not surprisingly demonstrated stronger conservation among more closely related mammalian species, and less conservation with non-mammalian vertebrates (Fig. 2 and data not shown). Although the long-standing dogma has been that key regulatory elements will show deep evolutionary conservation, recent analyses have often refuted this idea. While the recognition sites for some DNA binding factors are well-conserved across species [55], many are poorly conserved even within mammals, and less so in lower vertebrates [56], [57]. Genomic imprinting is a uniquely mammalian phenomenon, however, and while *Dlk1* is present in many non-mammalian vertebrates, it is not imprinted in these species. For genes subject to genomic imprinting, it is possible that constraint against harmful loss of imprinting would drive stronger conservation within mammals.

Within the 41 kb *Dlk1* upstream region of *127H5*, five highly conserved elements were able to function as enhancers in cell culture assays (Fig. 3), and two of these elements (CE4 and CE8) directed reproducible expression of a *lacZ* reporter transgene in a subset of *Dlk1*-expressing tissues in the mouse embryo (Fig. 4, 5). These data suggest that CE4 and CE8 contain *cis*-regulatory elements that direct *Dlk1* expression *in vivo*. *CE4-lacZ* and *CE8-lacZ* were both expressed in regions of the developing skeletal muscle, and the developing cartilage of the axial skeleton, particularly in the ribs and intervertebral discs (Fig. 4, 5). These patterns resemble the high level expression of endogenous *Dlk1* found in the developing skeletal muscle and the ossifying skeleton [27], [33]. Endogenous *Dlk1* is also expressed at high levels in many neuroendocrine cell types, including the pituitary somatotrophs, the chromaffin cells of the adrenal medulla, and the beta cells of the pancreas. The *127H5* transgene directed pituitary and adrenal expression (pancreas was not analyzed), and *CE8-lacZ* expressed *lacZ* in the chromaffin cells of the adrenal gland (Fig. 1, 5). Neither *CE4-lacZ* nor *CE8-lacZ* conferred expression in the pituitary, however, and no expression was seen from the *lacZ* transgenes in the developing pancreas. As *127H5* did not fully recapitulate endogenous *Dlk1* expression, and the sum of *CE4-lacZ* and *CE8-lacZ* did not fully recapitulate *127H5* expression, there remain *Dlk1* regulatory elements to be identified both within the span of *127H5*, and in sequences outside this region.

### Multiple Elements Regulate Dlk1 Expression in Developing Skeletal Muscle

In the late gestation embryo, *Dlk1* is most strongly expressed in developing skeletal muscle, but the gene is silenced in muscle shortly after birth. Sheep carrying the naturally-occurring Callipyge (*Clpg*) mutation fail to down-regulate *Dlk1* in specific muscle groups, resulting in increased skeletal muscle mass [58]–[61]. *Clpg* has been mapped to a point mutation in the *Dlk1*-*Meg3* intergenic region that is proposed to function as a cis-acting silencer in postnatal tissues [62]. In myoblasts, as in many other developing tissues, *Dlk1* may function to regulate the switch from a proliferating, immature cell population to a differentiating, mature one. In adult mice, *Dlk1* is reactivated in regenerating skeletal muscle, where it may be involved in satellite cell activation and/or proliferation [24].

MyoD is a key regulator of skeletal muscle development, where it transactivates muscle-specific genes through cis-acting E-box elements [63]. The loss of *Dlk1* in skeletal muscle-specific knockout mice is associated with reduced MyoD expression, perhaps through activation of the NF-kappa B pathway, providing support for an interaction between Dlk1 and MyoD in skeletal muscle [24]. The *CE4-lacZ* and *CE8-lacZ* transgenes showed strong myoblast expression (Fig. 4, 5), indicating that muscle-specific transcription factors may recognize the CE4 and CE8 elements. This work significantly narrows the regions to be investigated for possible interaction with MyoD and other, yet unknown, regulators of *Dlk1* tissue-expression in the mouse embryo.
